# Are migratory behaviours of bats socially transmitted?

**DOI:** 10.1098/rsos.150658

**Published:** 2016-04-06

**Authors:** E. F. Baerwald, R. M. R. Barclay

**Affiliations:** Department of Biological Sciences, University of Calgary, Calgary, Alberta, CanadaT2N 1N4

**Keywords:** hoary bat, *Lasiurus cinereus*, *Lasionycteris noctivagans*, migration, relatedness, silver-haired bat

## Abstract

To migrate, animals rely on endogenous, genetically inherited programmes, or socially transmitted information about routes and behaviours, or a combination of the two. In long-lived animals with extended parental care, as in bats, migration tends to be socially transmitted rather than endogenous. For a young bat to learn migration via social transmission, they would need to follow an experienced individual, most likely one roosting nearby. Therefore, we predicted that bats travelling together originate from the same place. It is also likely that young bats would follow their mothers or other kin, so we predicted that bats travelling together are more closely related to each other than bats not travelling together. To test our predictions, we used microsatellite genotypes and stable isotope values of *δ*^13^C, *δ*^15^N and *δ*^2^H to analyse the relatedness and geographical origins of migrating hoary bats (*Lasiurus cinereus/Aeorestes cinereus* (Baird *et al.* 2015 *J. Mammal*. 96, 1255–1274 (doi:10.1093/jmammal/gyv135)); *n* = 133) and silver-haired bats (*Lasionycteris noctivagans*; *n* = 87) killed at wind turbines over two consecutive autumn migrations. Contrary to our predictions, there was no evidence that related dyads of hoary bats or silver-haired bats were killed on the same night more frequently than expected by chance, or that the number of days between the fatalities of dyad members was influenced by relatedness or latitude of origin. Our data suggest that these bats do not socially transmit migration routes and behaviours among close kin.

## 1. Introduction

How animals find their way during migration has long fascinated us and inspired centuries of research. Migratory animals rely on endogenous, genetically inherited programmes or socially transmitted information about routes and behaviours, or a combination of the two [1–4]. The degree to which each source of information is used depends on numerous factors, including the age, experience, personality and sociality of an individual [5]. Thus, social transmission of migratory information may be more likely to occur in animals that live in groups that contain a mix of experienced leaders and naive followers than in solitary animals or in a group containing only experienced or only naive individuals.

Navigational abilities of experienced individuals are superior to those of inexperienced individuals [6,7]. In fact, following an experienced leader may be necessary for successful migration, as shown in the ultralight-led migrations of various species of birds [8,9]. Often, the leaders are parents guiding their offspring during their first migration, as in mammals such as some whales [10–12] and ungulates [13–15]. However, the experienced individual may not always be a parent [16].

Animals that learn components of migration from social transmission show increased flexibility and adaptability of migratory behaviour and a decreased use of suboptimal routes relative to those relying on genetic programmes [17]. For example, in whooping cranes (*Grus americana*), social learning reduces suboptimal migration strategies; inexperienced birds that followed older birds were significantly better at accurately arriving at their over-wintering location compared with inexperienced birds that flew only with other inexperienced birds [18].

The degree to which social transmission of migratory information occurs is correlated with life history. In long-lived birds with extended parental care, such as cranes, geese, swans, storks and bustards, migration tends to be socially transmitted, while it is under endogenous control in the majority of small songbirds [3,19]. Long lifespans facilitate the transmission of historic traditions, in African elephants (*Loxodonta africana*) for example [20,21].

Social transmission of migratory information and the use of optimal migration routes may be particularly important for animals with slow life histories because they have low reproductive rates, increasing the importance of successful migrations of juveniles. Migration is a risky behaviour, with most of the annual mortality occurring during migration [22–24], particularly in first-time migrants [25–27].

Bats have slow life histories and long lives [28], and we thus predict that social transmission of migratory behaviour is common in bats. However, we know little about their migratory behaviours. Bats are certainly capable of social transmission of behaviour [29–35]. Mothers and offspring are also capable of recognizing each other acoustically, even in large maternity colonies [36–38], which suggests that bonds occur between mother and offspring. There is also evidence of extended mother–pup associations [39–41]. Given that maternal investment is pronounced in bats, we hypothesized that migratory bats transmit information about migratory routes and behaviours to juveniles, as occurs in other animals with similar levels of parental investment.

Although highly gregarious species of bat likely migrate as a group (e.g. Mexican free-tailed bats (*Tadarida brasiliensis*) [42] and straw-coloured fruit bats (*Eidolon helvum*) [43]), it is not known how many species of bats flock while migrating. There are historical accounts of flocks of bats migrating during the day [44–47]. One of those flocks consisted of at least three sizes of bats, but the flock did not behave like flocks in the classic sense (i.e. with coordinated movements), but rather, appeared to be a group of individuals moving through the same place at the same time [45]. Echolocation activity and bat fatalities at wind energy facilities also appear in waves [48], but it is unclear whether this represents flocks in the classic sense, or whether multiple individuals simply take advantage of the same favourable environmental conditions to migrate (e.g. in low wind speeds) [48]. Regardless, flying in a group would allow for social transmission of migratory behaviours among both con- and heterospecifics [32].

Social transmission is most likely to occur from mother to offspring, but perhaps also from other conspecifics. For a young bat to learn migration via social transmission, it would need to follow an experienced individual, most likely one roosting nearby. Therefore, we predicted that bats travelling together on the same night originate from the same place. Stable isotopes can be used to determine the geographical origins of bat fur grown on the summering grounds, because fur is metabolically inert once grown and the stable isotope ratios in fur therefore reflect the environmental conditions (i.e. isoscape) where it was grown [49–51].

It is likely that young bats follow their mothers or other close relatives, so we predicted that bats travelling together on the same night are more closely related to each other than bats travelling on different nights. This pattern should be particularly apparent in adult females and juveniles because they roost together in family groups, whereas males roost independently. Given that some species of migratory bats are solitary (e.g. hoary bats; *Lasiurus cinereus/Aeorestes cinereus* [52]) and some are colonial (e.g. silver-haired bats; *Lasionycteris noctivagans*), we hypothesized that the degree to which migration is socially transmitted differs among species, being more prevalent in colonial than in solitary bats. If so, then we predicted that the similarity in geographical origins and the degree of relatedness of individuals travelling on the same night would be greater in colonial bats than in solitary ones, if colony members are closely related, as in some species such as big brown bats (*Eptesicus fuscus*) [53]. However, if colony members are not closely related, as in brown long-eared bats (*Plecotus auritus*) [54], then we might expect the degree of relatedness between migrating dyads to be less than we see in solitary bats, because young bats may have the opportunity to follow unrelated group members. To test our predictions, we used recently developed multilocus microsatellite genotypes [55] and stable isotope values of carbon (*δ*^13^C), nitrogen (*δ*^15^N) and hydrogen (*δ*^2^H) to analyse the temporal relatedness and geographical origins of migrating hoary bats and silver-haired bats killed at a wind energy facility in southwestern Alberta, Canada over two consecutive autumn migrations.

### 1.1. Study species

Hoary bats roost solitarily in tree foliage throughout much of North and South America [56]. Although we still do not understand the movement patterns or seasonal distributions of hoary bats, it seems they winter in Mexico and the southern United States, then migrate long distances north and east in the spring [51,57,58]. In summer, hoary bats in Canada are found throughout the prairies, aspen parkland and the southern boreal forests [50,59]. Reproductive females exhibit some degree of year-to-year site fidelity [60]. There may be some sexual segregation of hoary bats during summer, with females potentially migrating further than males [57,58,61] and males roosting separately from mother–pup family units [60], but stable isotope data show no strong evidence for sexual segregation at our site in southwestern Alberta [50]. However, the timing of fatalities suggests differential migration: adult males arrived one–two weeks before the adult females and subadults, which we first detected on the same night in both years [48]. Mating may occur in autumn during migration or on the wintering grounds, with one to four, but usually two pups born the following spring/early summer [56,62]. Pups are volant at about four weeks of age, but not weaned until about seven weeks of age [60].

Known wintering grounds of silver-haired bats are in the US Pacific Northwest, southwestern states and middle latitudes of the eastern United States [58,63]. The limited data on their migratory patterns suggest that in spring, silver-haired bats from the east migrate long distances north and east and western silver-haired bats migrate northward [58,64]. In summer, silver-haired bats in Canada are common throughout forested areas, where males roost singly and reproductive females form small colonies in tree cavities [65–67]. There is some sexual segregation during summer, with females potentially migrating farther than males [58], but stable isotope analysis of the fur of fatalities at turbines in southern Alberta showed no evidence of sexual segregation [50]. The timing of fatalities does not suggest differential migration, because adult males, adult females and subadults arrived together [48]. Mating appears to occur in autumn during migration and one or two, but usually two, pups are born the following spring/early summer [62,65]. Lactation lasts about five weeks and volancy appears to occur at about 4 weeks [65] (E. F. Baerwald and B. J. Klüg-Baerwald 2010, unpublished data).

## 2. Methods

### 2.1. Sample collection

We collected hair and skin samples from bat carcasses found under wind turbines in southwestern Alberta, Canada (49° 35′04′′ N, 113° 47′48′′ W). We searched for bat carcasses from 15 July to 30 September 2006 and 2007. We searched 10 randomly chosen turbines every day and the remaining 29 turbines once a week (see [68] for details). Searcher efficiency was very high (97% at turbines searched daily and 78% at those searched weekly [68]), thus bats killed by any of the 39 turbines had a high probability of being recovered within a week. For each carcass, we recorded species, age [69] and sex (when possible), and the degree of decomposition. In 2007, we verified age classifications during necropsies by noting the presence or absence of a thymus gland, which is present in subadults but absent in adults [70,71]. Details regarding fatalities can be found in [48].

We collected skin tissue from the wingtips of freshly killed bat carcasses (i.e. those killed the previous night at any of the 39 turbines) using dissecting scissors. Tissue was stored in 70–90% ethanol and kept in a −20°C freezer until DNA extraction. We collected hair from between the scapulae of bats killed the previous night, placed samples into plastic microcentrifuge tubes and stored them in a freezer until analysis.

### 2.2. DNA extraction, amplification and genotyping

Development of primers and microsatellite analysis were conducted at the University of Georgia's Savannah River Ecology Laboratory (see [55] for details on primer development). DNA was extracted using Qiagen DNEasy blood and tissue kit. PCR amplifications were performed in a 12.5 µl volume (10 mM Tris pH 8.4, 50 mM KCl, 25.0 µg ml^−1^ BSA, 0.4 µM unlabelled primer, 0.04 µM tag-labelled primer, 0.36 µM universal dye-labelled primer, 3.0 mM MgCl_2_, 0.8 mM dNTPs, 0.5 units AmpliTaq Gold^®^ Polymerase (Applied Biosystems) and 20 ng DNA template) using an Applied Biosystems GeneAmp 9700. Touchdown thermal cycling programmes [72] encompassing a 10°C span of annealing temperatures ranging between 65 and 55°C (TD65) were used for all loci. Touchdown cycling parameters consisted of an initial denaturation step of 5 min at 95°C followed by 20 cycles of 95°C for 30 s, highest annealing temperature (decreased 0.5°C per cycle) for 30 s, and 72°C for 30 s; and 20 cycles of 95°C for 30 s, lowest annealing temperature for 30 s, and 72°C for 30 s and a final extension at 72°C for 5 min. PCR products were run on an ABI-3130xl sequencer and sized with Naurox size-standard prepared as described in [73], except that unlabelled primers started with GTTT. Results were analysed using Genemapper v.~3.7 (Applied Biosystems).

One hundred and thirty-three hoary bats were genotyped at 19 microsatellite loci and 87 silver-haired bats were genotyped at 18 microsatellite loci. We calculated the number of alleles (*N*_a_), observed (*H*_o_) and expected heterozygosity (*H*_e_) and linkage disequilibrium (LD) using Arlequin v.~3.5.1.3 [74]. We used Micro-Checker v.~2.2.3 [75] to search for loci with the following genotyping errors: dropout of large alleles, stuttering and null alleles. We did not remove null alleles from further analysis, because the software programs we used are equipped to handle them.

### 2.3. Stable isotope analysis

Stable carbon (*δ*^13^C), nitrogen (*δ*^15^N) and deuterium (*δ*^2^H) analyses were conducted at the Saskatchewan Isotope Laboratory in the Department of Geological Sciences, University of Saskatchewan, Saskatoon, Saskatchewan, Canada (see [50] for details).

### 2.4. Analysis of relatedness

We examined relatedness of individual bats in multiple ways. First, we used the full-likelihood method of Colony v.~2.0.5.8 [76] to putatively distinguish dyads as unrelated, half-sibs, full-sibs or parent–offspring, from the genotypes of potential offspring and the potential mothers and fathers. We considered all young-of-the-year (from both 2006 and 2007) to be potential offspring and, given that bats have overlapping generations, we also considered adults from 2007 to be potential offspring of 2006 and 2007 adults (i.e. a 2007 adult could be the offspring of an older 2006 or 2007 adult). We considered all adult females and males to be potential mothers or fathers, respectively, each with a 0.5 probability of parenting one of the candidate offspring. To be classified as belonging to one of the relatedness categories, dyads had to have a greater than or equal to 95% probability of belonging to that category. We used the genotyping error estimated by Colony, assumed polygamy for both sexes, re-calculated allele frequencies and used no sibship prior (i.e. we made no assumptions about the average paternal or maternal sibship size).

We used Coancestry v. 1.0.1.5 [77] to calculate pairwise relatedness of all possible dyads. Coancestry calculates seven measures of relatedness simultaneously, but only the two maximum-likelihood methods (TrioML and DyadML) allow one to incorporate genotyping errors, which, if not accounted for, can affect the precision of the relatedness estimates. We used the genotyping errors calculated by Colony during the first analysis and compared the results of TrioML and DyadML to determine which model to use. For both species, the two models were highly correlated (*r*^2^ > 0.90), but the relatedness values were slightly higher in DyadML, so we used these values. However, the estimates from maximum-likelihood methods are not normally distributed, because they are bounded between zero and one, and thus result in loss of statistical power. Therefore, we used the package Related v. 1.0 in R 3.2.3 [78] to compare relatedness estimators and included the Wang estimator [79] in future Coancestry-based analyses because it performed the best (based on having the highest Pearson's correlation value greater than 0.90 for both species [78]). A relatedness of 0.25 is indicative of half-siblings, but to minimize the likelihood of making a type I error, we considered a dyad to be related if its relatedness was greater than or equal to 0.20.

### 2.5. Statistical analyses

To investigate whether related individuals were killed on the same night more frequently than expected by chance, we compared related and unrelated dyads killed on the same night to related and unrelated dyads killed on different nights using two different methods. For the putative relatedness categories of Colony, we used two-tailed Fisher's exact tests and for the relatedness values of Coancestry, we used two-tailed *t*-tests. We restricted the analysis to within-year dyads (i.e. we did not include dyads that contained individuals from different years). We used an α-value of 0.05. We present mean ± standard error where applicable.

We also examined whether the time between individuals within a dyad (in days) was influenced by their relatedness or the similarity in their geographical origins by using generalized linear models (GLMs) in JMP 10 (SAS Institute, Cary, NC). We used the normally distributed Wang relatedness value from the Coancestry analysis as a predictor variable. As a proxy for geographical origins, we used the absolute difference in the stable isotope values of *δ*^13^C, *δ*^15^N and *δ*^2^H between members of each dyad that had stable isotope values available for both individuals (hoary bats, *n* = 91 individuals and 2063 dyads; silver-haired bats, *n* = 59 individuals and 1711 dyads) as the other predictor variables. We included the interactions between relatedness and each of the isotope variables, but removed the interactions from the model if they were not significant. For the GLM, we used the log link function for Poisson distributions, accounted for overdispersion, and used an *α*-value of 0.05.

## 3. Results

### 3.1. Genetic diversity

For hoary bats, the number of alleles per locus ranged from three to 66 (electronic supplementary material, table S1). Average observed heterozygosity was 0.73 ± 0.23. Ten loci contained null alleles (electronic supplementary material, table S1). Forty-four of 171 comparisons (25.73%) showed evidence of significant LD. In silver-haired bats, the number of alleles per locus ranged from six to 20 (electronic supplementary material, table S2). Average observed heterozygosity was 0.67 ± 0.17. Eight loci contained null alleles (electronic supplementary material, table S2). Thirty-seven of 153 comparisons (24.18%) showed evidence of significant LD.

### 3.2. Stable isotope analysis

Details regarding overall patterns and relationships of the stable isotopes can be found in [50].

### 3.3. Statistical analyses

For hoary bats, Colony identified 134 related dyads, 83 of which occurred within the same year. The majority of putatively related dyads (97%) were half-sibling dyads, but there were two full-sibling dyads, one mother–offspring dyad and one father–offspring dyad. Neither the full-sibling dyads nor the parent–offspring dyads involved individuals killed on the same night. There was no evidence that a greater number of related dyads of hoary bats were killed on the same night more frequently than expected by chance (table 1).

**Table 1.** Contingency table based on relatedness categories of hoary bats (*Lasiurus cinereus*) as determined by Colony (Fisher's exact test, two-tailed, *p* = 1.0).

	related dyads	unrelated dyads
dyads killed on the same night	8	457
dyads killed on different nights	75	3888

Using the DyadML estimator, Coancestry identified 101 related dyads of hoary bats, 64 of which occurred within the same year, and using the Wang estimator, it identified 67 related dyads, 31 of which occurred within the same year. There was no evidence that mean relatedness of dyads of hoary bats killed on the same night was greater than that of dyads killed on different nights, regardless of the estimator used to determine related dyads (DyadML mean relatedness same night = 0.03 ± 0.002, mean relatedness different night = 0.03 ± 0.0008, two-tailed *t*-test, *t*_4352_ = −1.18, *p* = 0.24; Wang mean relatedness same night = −0.02 ± 0.001, mean relatedness different night = −0.03 ± 0.003, two-tailed *t*-test, *t*_4352_ = −1.9, *p* = 0.06).

In the GLM that assessed the influence of relatedness and geographical origin on the days between individuals in dyads of hoary bats, the model explained a significant proportion of the variation (model χ^2^/d.f._5_ = 27.05, *p* < 0.001; Pearson's χ^2^/d.f._2061_ = 12 768.07, *p* < 0.001; overdispersion = 6.20). The number of days between the fatalities of dyad members increased as the difference in their *δ*^13^C values increased, and as the difference in their *δ*^15^N values decreased, and was influenced by the interaction between dyad relatedness and the difference in their *δ*^2^H values (table 2); the number of days between fatalities was influenced by the similarity of their *δ*^2^H values, but when relatedness increased, this relationship decreased (table 2).

**Table 2.** Results of the generalized linear model that assessed the influence of relatedness and geographical origin (as determined by stable isotope values) of hoary bats (*Lasiurus cinereus*) on the number of days between fatalities of dyad members. Wang relatedness values are from Coancestry and based on the relatedness estimator by Wang [79]. Values in italics are significant at α = 0.05.

parameter	estimate	s.e.	*χ*^2^	*p*-value
intercept	2.136	0.047	1660.15	*<0.001*
Wang relatedness	−0.706	0.270	7.04	*0.008*
difference in *δ*^13^C	0.045	0.019	5.46	*0.02*
difference in *δ*^15^N	−0.049	0.024	4.11	*0.04*
difference in *δ*^2^H	−0.003	0.001	8.27	*0.004*
Wang relatedness × difference in *δ*^2^H	−0.028	0.013	4.84	*0.03*

For silver-haired bats, Colony identified 96 related dyads, 58 of which occurred within the same year. The majority of putatively related dyads (98%) were half-sibling dyads, but there was one full-sibling dyad and one father–offspring dyad. Neither the full-sibling dyad nor the father–offspring dyad involved individuals killed on the same night. There was no evidence that a greater number of related dyads of silver-haired bats were killed on the same night more frequently than expected by chance (table 3).

**Table 3.** Contingency table of relatedness categories of dyads of silver-haired bats (*Lasionycteris noctivagans*) as determined by Colony (Fisher's exact test, two-tailed, *p*** **=** **0.06).

	related dyads	unrelated dyads
dyads killed on the same night	9	147
dyads killed on different nights	49	1644

Using the DyadML estimator, Coancestry identified 155 related dyads of silver-haired bats, 113 of which occurred within the same year, and using the Wang estimator, it identified 53 related dyads, 40 of which occurred within the same year. There was no evidence that mean relatedness of dyads of silver-haired bats killed on the same night was greater than that of dyads killed on different nights, regardless of the estimator used to determine related dyads (DyadML mean relatedness same night = 0.04 ± 0.005, mean relatedness different night = 0.04 ± 0.001, two-tailed *t*-test, *t*_2853_ = 0.22, *p* = 0.83; Wang mean relatedness same night = −0.05 ± 0.009, mean relatedness different night = −0.05 ± 0.002, two-tailed *t*-test, *t*_2853_ = −0.74, *p* = 0.46).

In the GLM that assessed the influence of relatedness and geographical origin on the days between individuals in dyads of silver-haired bats, the model explained a significant proportion of the variation (model *χ*^2^/d.f._4_ = 19.7, *p* < 0.001; Pearson's *χ*^2^/d.f._842_ = 7891.40, *p* < 0.001; overdispersion = 9.72). The number of days between the fatalities of dyad members increased as the difference in their *δ*^15^N values increased but was not influenced by any other predictor variable (table 4).

**Table 4.** Results of the generalized linear model that assessed the influence of relatedness and geographical origin (as determined by stable isotope values) of silver-haired bats (*Lasionycteris noctivagans*) on the number of days between fatalities of dyad members. Wang relatedness values are from Coancestry and based on the relatedness estimator by Wang [79]. Values in italics are significant at *α*** **=** **0.05.

parameter	estimate	s.e.	*χ*^2^	*p*-value
intercept	2.38	0.072	835.43	*<0.001*
Wang relatedness	−0.074	0.279	0.07	0.79
difference in *δ*^13^C	−0.019	0.035	0.29	0.58
difference in *δ*^15^N	0.106	0.024	18.91	*<0.001*
difference in *δ*^2^H	−0.002	0.002	1.65	0.20

## 4. Discussion

Contrary to our predictions, we found no conclusive evidence that either hoary bats or silver-haired bats migrate in family groups. In both species, the majority of dyads were not related and although a few related dyads were killed on the same night, 85–93% of the related dyads were killed on different nights. None of the putative parent–offspring (*n* = 1) or full-sibling dyads (*n* = 3) were killed on the same night. However, siblings from the same litter may have multiple paternity and be half-siblings rather than full-siblings, as seen in other species of bat that have twins [80–82], thus obscuring the closeness of the family connections. Thus, it appears that young bats on their first migrations are not following their mothers or travelling with their sibling(s). Given that sexual segregation occurs on the summering grounds for both species, it is also highly unlikely that juvenile bats are following their fathers. In silver-haired bats, young bats may follow non-related colony members, but hoary bats do not live in colonies, so do not have other group members to follow.

If inexperienced bats are not following kin or colony members, then they may follow other conspecifics. However, our hypothesis that bats migrating on the same night originate from a similar location was also not fully supported. In general, *δ*^2^H decreases with increasing latitude and elevation and this pattern is linked to the *δ*^2^H in precipitation [83,84]. *δ*^13^C and *δ*^15^N values also decrease with increasing latitude and elevation, but this is linked to climate and habitat shifts that occur with increasing latitude, such as shifts to cooler, drier conditions and from predominantly C_4_ to C_3_ plants [85–90]. Therefore, it appears that bats travelling on the same night originate from similar habitats, as indicated by the significant effect of *δ*^13^C and *δ*^15^N for hoary bats and *δ*^15^N for silver-haired bats, but not necessarily from similar latitudes, as a significant effect of *δ*^2^H would have indicated [50]. Instead, the negative interaction between relatedness of hoary bats and *δ*^2^H indicates that the correlation of *δ*^2^H with the number of days between dyad members is diminished as relatedness increases. This may suggest a subtle impact of relatedness on timing, but we cannot be certain given this dataset. Our stable isotope results may well suggest that individuals travelling together on the same night are not doing so in a coordinated manner, but rather, responding to similar cues in similar habitats and moving accordingly.

In addition to our data indicating that neither silver-haired bats nor hoary bats are migrating in family groups, there is evidence from other species of bats that mothers may embark on autumn movements before their young do [91–93]. This is surprising given the slow life histories of bats and the high mortality commonly associated with the first migration of juvenile animals [25]. Although mortality rates of juvenile bats during their first migration are not known, migratory bats in the genera *Perimyotis, Nyctalus*, *Lasiurus* and *Lasionycteris* commonly have twins, which is unusual among bats [28]. This, and the fact that juvenile hoary bats and silver-haired bats of both sexes are ready to mate during their first autumn, which is also unusual among bats [62], suggests that mortality of first-year individuals is relatively high. Survival estimates of migratory Leisler's bat (*Nyctalus leisleri*) are relatively high for adults (annual survival estimates for adult females vary from 0.45 to 0.61 and from 0.55 to 0.91 for adult males [94,95]), but are lower for first-year individuals, with annual survival estimates of 0.45 for juvenile females [94].

Because information obtained socially is sometimes easier to obtain, but less reliable than information obtained asocially (i.e. through trial-and-error), there is often a mixture within a group in the number of individuals acquiring information asocially and those acquiring information socially [96,97]. Information should be obtained socially when the costs of independently acquiring that information outweigh the benefits (‘copy when asocial learning is costly’ [96]) or when the independently acquired information results in too much uncertainty (‘copy when uncertain’ [96–99]). We assumed that both of these criteria were fulfilled and that the benefit of increasing the probability of a successful first migration of offspring would outweigh any costs a mother incurred from travelling with the pups. However, there are multiple costs and benefits that affect the decision to lead, and also to follow.

The costs and benefits of social transmission of migratory information can be divided into two categories, those associated with being in a group and those associated with the teaching or learning of behaviours. Benefits of being in a group may include increased predator detection and social thermoregulation, whereas costs may include increased conspicuousness and competition for resources [100,101]. Because these costs and benefits are related to being in a group, they should be shared among group members (e.g. among leaders and followers), although not necessarily equally. It may be that for reproductive female hoary bats, the costs of travelling in a group outweigh any benefits and they leave their offspring, becoming solitary again during migration, thus precluding social transmission of migratory behaviours from mother to offspring.

If the social transmission of migratory information is purposeful (i.e. migratory behaviours are taught), then it is likely that the costs are higher for the leaders/demonstrators than for the followers/observers. In fact, one of the main characteristics of teaching behaviour is that the~demonstrator incurs a cost, or at least not an immediate benefit, from altering their behaviour in the presence of a naive observer [102]. Costs may be measured in time, energy and mortality [103]. We know virtually nothing about the importance of migratory timing to bats, but if timing of departure and arrival and/or minimization of travel time is important, and travelling in a group with naive individuals negatively affects timing for experienced individuals, then experienced individuals may forego social transmission. For example, diets of adult and juvenile bats frequently differ [104–106], as found in the specific individual bats used in our study [107]. If the dietary differences result in longer foraging times for juveniles than for adults, and this slows migratory movement, this delay may be overly costly for adults.

We also know little about the mating behaviour of hoary or silver-haired bats, but as in other temperate-zone bats, they likely mate in autumn, during migration [56,62,65]. Mating may occur along migration routes, potentially with males intercepting females at lekking sites [108], although there is limited evidence to support this. Juveniles of both species may be ready to mate during their first migration, although there is no evidence they successfully do so [62]. Regardless, if migrating with juveniles negatively affects mating opportunities for the mothers, perhaps by affecting timing, they may forego social transmission.

Bats may employ a ‘fly-and-forage strategy’, foraging periodically during migration flights rather than foraging solely at emergence in the evening, as seen in Nathusius' pipistrelle (*Pipistrellus nathusii*) [109]. If attention to other individuals' echolocation is required to maintain contact with group members, and if this involves a trade-off between attention and foraging, the costs of attention may be too high, thus favouring solitary migration. It may be particularly challenging to maintain contact among small-bodied individuals travelling through the vast aerosphere, especially at night. Although bats may use echolocation [110,111] and passerines may use flight calls to communicate with group members [112,113], the range of these vocalizations may be insufficient to ensure group cohesion (but see [114]). Perhaps this constraint partially explains why migration in the majority of nocturnally migrating songbirds is under endogenous control rather than learned through social transmission [3,19].

If migratory behaviours in bats are the result of a genetic programme rather than social transmission, how do individuals find their way? The literature on orientation and navigation systems of animals during migration is vast and rich, particularly for birds [115–118]. Bats are capable of perceiving stars [119] and using post-sunset glow [120,121], the Earth's magnetic field [121–123] and geographical landmarks and linear features [124–127] for orientation and the creation of large-scale navigational maps [128]. Although bats are highly specialized for echolocation, it seems unlikely that they use echolocation to navigate over long distances [129]. They and other echolocating animals, such as porpoises [130], use echolocation for spatial orientation at small/local scales [131] and not for long-distance movements.

We hypothesized that the benefits of social transmission, such as increased flexibility and decreased use of suboptimal routes [17], would outweigh any costs associated with travelling in a group or teaching, but our findings suggest otherwise. This leads to many interesting questions and areas for further research. Why do bats not rely on social transmission of migratory behaviours as in other migratory mammals? How do bats, particularly juvenile bats, find their way to their over-wintering grounds? Do bats travel in groups of unrelated individuals, and if so, how do they maintain cohesion? Is social transmission more likely in the nomadic migrations of nectarivores such as the grey-headed flying-fox (*Pteropus poliocephalus*) that follow the flowering of plants [132,133]? What is the mortality rate of juvenile bats during their first migration? How do these mortality rates differ with social structure, roosting ecology and migration distance? In short, much more work is needed on the use and relative importance of social transmission and endogenous programmes to bats before we can fully understand their migration biology.

## Supplementary Material

Molecular diversity indices for 19 microsatellite loci for hoary bast (Lasiurus cinereus) and 18 microsatellite loci for silver-haired bats (Lasionycteris noctivagans).
