# Supplementary material for: Are migratory behaviours of bats socially transmitted?
Source: R Soc Open Sci. 2016 Apr 6;3(4):150658. doi: 10.1098/rsos.150658 (PMC4852631; doi:10.1098/rsos.150658)
Supplement: Molecular diversity indices for 19 microsatellite loci for hoary bast (Lasiurus cinereus) and 18 microsatellite loci for silver-haired bats (Lasionycteris noctivagans). [file rsos150658supp1.docx]

Table S1 Molecular diversity indices of 19 hoary bat (*Lasiurus cinereus*) microsatellite loci from 133 individuals. Frequency of null alleles is based on (1) and values in bold are loci that contain null alleles.

| Locus | Number of alleles | Number of genotypes | Observed heterozygosity | Expected heterozygosity | Frequency of null alleles |
| --- | --- | --- | --- | --- | --- |
| Laci1 | 65 | 130 | 0.762 | 0.972 | **0.107** |
| Laci15 | 11 | 130 | 0.831 | 0.826 | -0.007 |
| Laci20 | 9 | 131 | 0.779 | 0.771 | -0.006 |
| Laci22 | 19 | 131 | 0.901 | 0.865 | -0.024 |
| Laci25 | 10 | 132 | 0.273 | 0.786 | **0.313** |
| Laci26 | 28 | 130 | 0.938 | 0.910 | -0.018 |
| Laci28 | 3 | 132 | 0.015 | 0.015 | **0.109** |
| Laci29 | 19 | 129 | 0.674 | 0.867 | **0.109** |
| Laci31 | 18 | 132 | 0.879 | 0.887 | 0.002 |
| Laci33 | 23 | 132 | 0.856 | 0.884 | 0.014 |
| Laci34 | 23 | 131 | 0.863 | 0.934 | **0.037** |
| Laci35 | 66 | 132 | 0.894 | 0.977 | 0.041 |
| Laci36 | 21 | 132 | 0.894 | 0.882 | -0.008 |
| Laci38 | 39 | 131 | 0.534 | 0.946 | **0.217** |
| Laci39 | 27 | 132 | 0.856 | 0.872 | 0.005 |
| Laci41 | 33 | 131 | 0.771 | 0.941 | **0.088** |
| Laci42 | 12 | 131 | 0.634 | 0.835 | **0.119** |
| Laci43 | 17 | 132 | 0.689 | 0.878 | **0.105** |
| Laci48 | 24 | 132 | 0.750 | 0.868 | **0.066** |

Table S2 Molecular diversity indices of 18 silver-haired bat (*Lasionycteris noctivagans*) microsatellite loci. Frequency of null alleles is based on (1) and values in bold are loci that contain null alleles.

| Alberta Summerview  (n = 87) |  |  |  |  |  |
| --- | --- | --- | --- | --- | --- |
| Locus | Number of alleles | Number of genotypes | Observed heterozygosity | Expected heterozygosity | Frequency of null alleles |
| Lano1 | 7 | 87 | 0.644 | 0.709 | 0.05 |
| Lano3 | 8 | 87 | 0.862 | 0.796 | -0.05 |
| Lano4 | 11 | 86 | 0.488 | 0.843 | **0.21** |
| Lano8 | 13 | 85 | 0.800 | 0.858 | 0.03 |
| Lano10 | 17 | 87 | 0.655 | 0.867 | **0.12** |
| Lano13 | 14 | 87 | 0.828 | 0.880 | 0.03 |
| Lano15 | 10 | 84 | 0.750 | 0.767 | 0.01 |
| Lano22 | 18 | 86 | 0.907 | 0.869 | -0.03 |
| Lano26 | 12 | 87 | 0.345 | 0.829 | **0.29** |
| Lano28 | 6 | 86 | 0.674 | 0.671 | -0.01 |
| Lano29 | 12 | 87 | 0.816 | 0.862 | 0.03 |
| Lano31 | 14 | 85 | 0.471 | 0.850 | **0.22** |
| Lano32 | 12 | 86 | 0.547 | 0.840 | **0.17** |
| Lano34 | 13 | 87 | 0.701 | 0.857 | **0.09** |
| Lano35 | 20 | 87 | 0.782 | 0.910 | 0.07 |
| Lano40 | 17 | 87 | 0.805 | 0.817 | 0.00 |
| Lano41 | 10 | 83 | 0.386 | 0.813 | **0.25** |
| Lano44 | 19 | 86 | 0.535 | 0.917 | **0.21** |

1. Van Oosterhout C, Hutchinson WF, Wills DP, Shipley P. MICRO‐CHECKER: software for identifying and correcting genotyping errors in microsatellite data. Mol Ecol Notes. 2004;4(3):535-8.
